# Periodontal status among 12-year-old schoolchildren: a population-based cross-sectional study in Quito, Ecuador

**DOI:** 10.1590/1807-3107bor-2024.vol38.0002

**Published:** 2024-01-05

**Authors:** Marco MEDINA-VEGA, Mariela Cumandá Balseca IBARRA, Maritza Del Carmen QUEZADA-CONDE, Isabella Neme Ribeiro dos REIS, Antonio Carlos FRIAS, Daniela Prócida RAGGIO, Edgard MICHEL-CROSATO, Fausto Medeiros MENDES, Claudio Mendes PANNUTI, Giuseppe Alexandre ROMITO

**Affiliations:** (a) Central University of Ecuador, School of Dentistry, Quito, Ecuador.; (b) Universidade de São Paulo – USP, School of Dentistry, Department of Stomatology, São Paulo, SP, Brazil.; (c) Universidade de São Paulo – USP, School of Dentistry, Department of Pediatric Dentistry, São Paulo, SP, Brazil.; (d) Universidade de São Paulo – USP, School of Dentistry, Department of Community Dentistry, São Paulo, SP, Brazil.

**Keywords:** Gingivitis, Dental Calculus, Child, Epidemiology

## Abstract

This study aimed to estimate the prevalence and extent of bleeding on probing and calculus in 12-year-old schoolchildren of Quito, Ecuador, and evaluate the associated factors. We conducted an epidemiological survey with a representative sample of 1,100 12-year-old schoolchildren from public schools in the urban area of Quito, Ecuador. We assessed the periodontal health using the Community Periodontal Index (CPI). The prevalence and extent of the periodontal condition was based on the presence of at least one site with bleeding on probing (BOP), and the presence of dental calculus was also evaluated. We used univariate and multiple multilevel Poisson regression analyses to verify the association between the independent variables and the number of sextants with BOP and calculus. The prevalence of BOP and calculus was 92% and 69.9%, respectively. The adjusted mean of the affected sextants was 4.3 and 2.2 for BOP and calculus, respectively. The mother’s schooling and malocclusion were associated with the number of sextants with bleeding. The mother’s schooling and dental caries experience were associated with calculus. Gingival bleeding and the presence of dental calculus are highly prevalent in 12-year-old schoolchildren from Quito. Gingival bleeding is associated with maternal education and malocclusion, and dental calculus is associated with maternal education and dental caries.

## Introduction

Periodontal diseases are a major public health concern worldwide due to their high prevalence in both developed and developing countries.^1^ Despite extensive research conducted on the prevalence of these diseases, a significant proportion of the studies has focused on a global scale, which may hinder the development of effective action plans for specific regions or populations.^2^ In Latin America, few population-based studies investigated the periodontal conditions of its populations, particularly in children and adolescents. Moreover, despite its significance in the region, Quito - the capital city of Ecuador - lacks studies on the periodontal conditions of schoolchildren.

Gingivitis, defined as gingival bleeding in at least one site, is the most prevalent periodontal disease in children and adolescents. The most common type of gingivitis is induced by biofilm.^3,4^ Signs and symptoms of gingivitis are gingival redness, swelling, and bleeding without periodontal attachment loss.^5^ Gingival inflammation usually does not cause pain, and despite being a common finding in children^6^, most patients do not perceive the disease.^7^ The factors associated with the prevalence and extent of gingivitis go beyond biological determinants (i.e., dental biofilm) and can include local factors (dental calculus) and socioeconomic variables such as gender, education, and family income.^3,5,8^

A recent cross-sectional study assessed the prevalence, severity, and extent of clinical periodontal attachment loss in adolescents aged 15 to 19 in six Latin American cities, including 144 participants from Quito.^9^ The assessments were performed between 2010 and 2012. The prevalence of gingivitis ranged from 23 to 77%. Living in Quito increased the chance of having at least one site with clinical attachment loss (CAL) ≥ 3 mm, compared to living in other Latin American cities. However, even though this was a population-based study, the results were not presented according to city. Thus, it is not possible to estimate the distribution and determinants of periodontal disease in Quito. The samples from each center were not representative of each city individually. Thus, the periodontal condition of schoolchildren living in Quito deserves attention.

Given the scarcity of epidemiological surveys on the oral conditions and related factors of Ecuadorian schoolchildren, a collaborative project involving researchers from the University of São Paulo (FOUSP) and the Central University of Ecuador (UCE) was conducted. This study collected data on the prevalence of several oral health conditions of schoolchildren, such as gingival bleeding, calculus, dental caries, malocclusion, traumatic dental injuries, and fluorosis. Additionally, socioeconomic and demographic data were collected.

While descriptive results of the “QUITO Oral Health Survey” have been published,^10^ the extent of gingivitis and its determinants have not been reported yet. Thus, the aim of this paper was to describe the prevalence and extent of BOP and calculus among 12-year-old public school children in the urban area of Quito, Ecuador, and to evaluate the associated factors.

## Methodology

### Study design

We conducted a cross-sectional study with a representative population sample of 12-year-old schoolchildren from public schools in the urban area of Quito (Ecuador). The study aimed to assess the prevalence and extent of BOP and dental calculus using the CPI. This publication is part of the “QUITO Oral Health Survey” (QUITO-OH Survey), which assessed periodontal conditions, dental caries, dental trauma, malocclusion, and oral health-related quality of life.

We reported the present study according to the Strengthening the Reporting of Observational Studies in Epidemiology (STROBE) for cross-sectional studies.^11^

### Participants

The Institutional Review Boards of the University of São Paulo and the Central University of Ecuador approved the protocol. Parents or legal guardians of the schoolchildren were contacted and signed an informed consent. Further, schoolchildren signed an assent form before their inclusion.

One thousand one hundred 12-year-old students were randomly selected from public schools in the urban area of Quito, Ecuador. Information regarding the sampling method and sample size calculation can be found in a previous publication.^10^ Only children born between April 2004 and March 2005 were included. Children whose parents or guardians did not sign the consent form, who did not agree to participate, who present or report systemic diseases, and who did not attend school the days of the examinations were not included.

### Data collection and variables

The data collection period was from March to May 2017. Six investigators were divided into three groups, each consisting of two examiners, two individuals responsible for taking notes, one interviewer, and one assistant. The team received clinical and theoretical training, and an inter-examiner calibration was performed. Details about calibration were previously described.^10^ Inter-examiner reproducibility among the 6 examiners during the calibration was 92.2% (kappa value 0.73, 95%CI 0.65 to 0.82) and 92.5% (kappa value 0.85, 95%CI 0.79 to 0.89) for gingival bleeding and presence of calculus, respectively.

The dependent variable was prevalence and extent of BOP and calculus, while the independent variables included socioeconomic and demographic factors (such as sex, family income, parental education, and household size, as person per room), caries experience as measured by the DMF-T index, and malocclusion as measured by the DAI index.

We organized the groups according to the school’s geographic localization (Southern, Central, and Northern Quito). Information regarding access to dental services and demographic and socioeconomic data were collected through a structured questionnaire that was sent to the parents who had provided signed consent forms. The questionnaire included information on family income (which was converted to the Ecuadorian Minimum Wage), level of education for both parents (measured by years of regular study), number of individuals living in the household, and number of rooms in the home.

We performed a two-stage cluster sampling, in which the schools constituted the first stage and the children enrolled in these schools constituted the second stage.^10^ Every child was examined using the WHO CPI ball-point probe and sterilized flat mirrors under artificial light. When necessary, teeth were dried using gauze pads before the examination. The presence and extent of periodontal conditions were assessed using Community Periodontal Index (CPI).^12^ Examiners gently inserted a ball-point periodontal probe (less than 20g of force) in the sulcus of six sites per tooth (mesio-buccal, buccal, disto-buccal, disto-lingual, lingual, and mesio-lingual) of teeth 2, 8, 14, 19, 24, and 30. Regarding bleeding, each sextant was assigned a code: 0- no bleeding, 1- bleeding, X- tooth not presented, 9- tooth excluded. We used the same codes to record the presence of calculus. BOP and dental calculus conditions were independently evaluated so that the less severe injury was not underestimated. The examiners evaluated the sites for BOP (yes or no) and calculus (yes or no). We defined gingivitis as the presence of BOP in at least one site.^2^ The presence of periodontal pockets was not evaluated since this condition is not expected at this age.

Dental caries and malocclusion were also evaluated. We used the Decayed, Missed, and Filled permanent teeth index (DMF-T index) for dental caries evaluation, as proposed by WHO.^2^ Children were classified as having no caries (DMF-T = 0) and as having dental caries (DMF-T >0). Moreover, the DAI index was used for malocclusion evaluation.^13^ We categorized the scores according to predetermined cut-offs: a) normal occlusion; b) definite malocclusion; c) severe malocclusion; or d) handicapping malocclusion. For the present study, malocclusion was considered when the child was classified with severe or handicapping malocclusion.

### Statistical analysis

We entered data into a database (Excel 2018; Microsoft Corporation, Redmond, WA, USA) and performed statistical analyses using Stata^®^ 16.0 (Stata Corporation, College Station, USA).

The prevalence of the diseases studied was described using the absolute frequency and the respective 95% confidence interval (95%CI). We also calculated the prevalence weighted by the study design. We performed univariate multilevel Poisson regression analysis to verify the association between the individual independent variables (sex, family income, parental education, household size, caries experience, and malocclusion) and the prevalence and extent of bleeding on probing and calculus. Therefore, the association measure obtained was the rate ratio (RR). Then, we performed multiple Poisson regression analyses to verify the association between the independent variables and the number of sextants with BOP.

The presence of calculus was analyzed as a dichotomous variable. Therefore, we also conducted multilevel Poisson regression, but the association measure calculated was the prevalence ratios (PRs) and respective 95% CIs. We adopted a significance level of p < 0.05 for all statistical tests.

## RESULTS

Thirty-three schools from the urban zone of Quito were randomly selected, but only 31 school’s coordinators accepted to participate. We randomly selected 1,100 schoolchildren from the 31 schools. The positive response rate was 90.7%, with the inclusion of 998 schoolchildren. Non-participation occurred because of the following reasons: did not sign the consent form (96 schoolchildren), nonattendance on the examination day (4 schoolchildren), and refusal to be examined (2 schoolchildren).

The prevalence of BOP and calculus are presented in Table 1. The adjusted prevalence of BOP and calculus was 92.0% and 69.9%, respectively, and the mean number of sextants affected by BOP and calculus was 4.32 and 2.18, respectively. Descriptive analysis and univariate Poisson multilevel regression analysis regarding individual independent variables and the number of bleeding sextants are presented in Table 2. In the univariate analysis, mother’s schooling, father’s schooling, and malocclusion were associated with number of sextants with bleeding. The results of multivariate analyses are presented in Table 3. Schoolchildren who had severe or handicapping malocclusion had 10% more sextants with gingival bleeding than those who had normal occlusion (RR = 1.10, 95%CI 1.03–1.17, p = 0.003), and schoolchildren whose mothers had a higher education than primary education had 5% fewer sextants with BOP than those whose mothers had complete primary school (RR = 0.95, 95% CI 0.90–0.99, p = 0.018). In the multivariate model, father’s schooling was not significantly associated with the outcome, probably due to collinearity with mother’s schooling.

**Table 1:** Distribution of BOP and dental calculus.

Variable	BOP		Calculus	

	95%CI			
Unadjusted prevalence	0.932 (0.914–0.946)		0.735 (0.707–0.762)	
Prevalence adjusted by study design	0.920 (0.871–0.952)		0.699 (0.605–0.779)	
Mean of sextants affected	4.55 (4.43–4.67)		2.33 (2.21–2.45)	
Median (IQ) of sextants affected	5.0 (4.0 – 6.0)		2.0 (0.0 – 4.0)	
Mean of sextants affected, adjusted for study design	4.32 (3.91–4.73)		2.18 (1.82–2.55)	
Proportion by number of affected sextants	%	% accumulated	%	% accumulated
0	6.8	6.8	26.5	26.5
1	4.1	10.9	12.7	39.2
2	6.7	17.7	15.6	54.8
3	7.0	24.7	13.9	68.7
4	9.4	34.1	14.3	83.1
5	16.9	51.0	11.3	94.4
6	49.0	100.0	5.6	100.0

**Table 2:** Descriptive analysis and univariate Poisson multilevel regression analysis between individual independent variables and number of sextants with bleeding.

Independent variables	Number of sextants with BOP		Unadjusted RR	p-value

	n (%)	Mean (SD)	(95% CI)	
Sex (Ref.: female)	554 (55.5)	4.65 (1.88)	1.00	
Male	444 (44.5)	4.42 (1.93)	0.98 (0.93–1.03)	0.395
Family income (Ref.: Up to 1 EMW)	434 (45.2)	4.31 (1.96)	1.00	
More than 1 EMW	526 (54.8)	4.77 (1.82)	1.01 (0.97–1.05)	0.575
Mother’s schooling (Ref.: Up to 8 years of formal education)	343 (34.4)	4.66 (1.78)	1.00	
More than 8 years of formal education	654 (65.6)	4.49 (1.97)	0.94 (0.90–0.99)	0.021*
Father’s schooling (Ref.: Up to 8 years of formal education)	343 (34.5)	4.74 (1.70)	1.00	
More than 8 years of formal education	650 (65.5)	4.45 (2.00)	0.94 (0.90–0.99)	0.011*
Persons per room (Ref.: Up to 1.7 person per room)	603 (60.8)	4.45 (1.96)	1.00	
More than 1.7 person per room	389 (39.2)	4.70 (1.82)	1.02 (0.99–1.06)	0.127
Caries experience (REF.: DMF-T index = 0)	414 (41.5)	4.61 (1.87)	1.00	
DMF-T between 1 and 4	523 (52.4)	4.49 (1.93)	0.95 (0.91–1.00)	0.061
DMF-T greater than 4	61 (6.1)	4.66 (1.94)	1.01 (0.91–1.12)	0.807
Malocclusion (Ref.: Normal / Definitive malocclusion)	364 (36.5)	4.41 (2.06)	1.00	
Severe / Handicapping malocclusion	634 (63.5)	4.63 (1.81)	1.10 (1.03–1.17)	0.005*

EMW: Ecuadorian minimum wage at the time of data collection (U$ 375.00); DMF-T index: Number of decayed, missing or filled teeth; DAI: Dental Aesthetics Index; SD: standard deviation; RR: rate ratio; 95%CI:95% confidence interval. Some variables do not add up to 998 children due to unreported data.

**Table 3:** Multiple Poisson multilevel regression analysis of the association between exposure variables and the number of sextants with BOP.

Independent variables	Adjusted RR	95%CI	p-value
Gender			
Female	1.00		
Male	0.98	0.9–1.03	0.382
Mother’s schooling			
Up to 8 years of formal education	1.00		
More than 8 years of formal education	0.95	0.90–0.99	0.018*
Malocclusion			
Normal / Definitive DAI index	1.00		
Severe / Handicapping DAI index	1.10	1.03–1.17	0.003*

EMW: Ecuadorian minimum wage at the time of data collection (U$ 375.00); DAI: Dental Aesthetics Index; RR: rate ratio; 95%CI: 95% confidence interval.

The descriptive and univariate Poisson multilevel regression analysis regarding independent variables and presence of dental calculus (dichotomous variable) are presented in Table 4. Mother’s schooling and dental caries were associated with presence of calculus in the univariate analysis. In the multiple analysis (Table 5), schoolchildren whose mothers had a higher level of education than primary education had 14% lower prevalence of dental calculus (PR = 0.86, 95%CI 0.80–0.93, p = < 0.001). Furthermore, schoolchildren with DMF-T greater than 4 had 9% higher prevalence of dental calculus (PR = 1.09, 95%CI 1.00–1.19, p = 0.044).

**Table 4:** Descriptive analysis and univariate Poisson multilevel regression analysis between independent variables and presence of dental calculus (dichotomous variable).

Independent variables	Presence of calculus		Unadjusted prevalence ratio (RP) (95%CI)	p-value

	No (%)	Yes (%)		
Gender				
Female	141 (25.5)	413 (74.5)	1.00	
Male	123 (27.7)	321 (72.3)	0.98 (0.90 to 1.07)	0.701
Family income				
Up to 1 minimum wage [EMW]	132 (30.4)	302 (69.6)	1.00	
More than 1 minimum wage [EMW]	125 (23.8)	401 (76.2)	1.01 (0.92 to 1.12)	0.788
Mother’s schooling				
Up to 8 years of formal education	67 (19.5)	276 (80.5)	1.00	
More than 8 years of formal education	197 (30.1)	457 (69.9)	0.86 (0.80 to 0.93)	< 0.001*
Father’s schooling				
Up to 8 years of formal education	82 (23.9)	261 (76.1)	1.00	
More than 8 years of formal education	180 (27.7)	470 (72.3)	0.95 (0.88 to 1.02)	0.176
Persons per room				
Up to 1.7 people per room	172 (28.5)	431 (71.5)	1.00	
More than 1.7 people per room	92 (23.7)	297 (76.4)	1.05 (0.98 to 1.13)	0.177
Dental caries experience				
DMF-T index = 0	110 (26.6)	304 (73.4)	1.00	
DMF-T index between 1 and 4	145 (27.7)	378 (72.3)	0.97 (0.89 to 1.06)	0.513
DMF-T index greater than 4	9 (14.5)	734 (73.5)	1.11 (1.02 to 1.06)	0.014*
Malocclusion				
Normal / Definitive DAI index	87 (23.9)	277 (76.1)	1.00	
Severe / Handicapping DAI index	177 (27.9)	457 (72.1)	1.00 (0.93 to 1.08)	0.995

MW: Ecuadorian minimum wage at the time of data collection (U$ 375.00); DMF-T index: number of decayed, missing or filled teeth; DAI: Dental Aesthetics Index; PR: prevalence ratio; 95%CI: 95% confidence interval . Some variables do not add up to 998 children due to unreported data.

**Table 5:** Multiple Poisson multilevel regression analysis of the association between exposure variables and the presence of calculus.

Independent variables	Adjusted prevalence ratio (RP)	95%CI	p-value
Gender			
Female	1.00		
Male	0.99	0.91–1.08	0.788
Mother’s schooling			
Up to 8 years of formal education	1.00		
More than 8 years of formal education	0.86	0.80–0.93	< 0.001*
Dental caries experience			
DMF-T index = 0	1.00		
DMF-T index between 1 and 4	0.96	0.89–1.05	0.378
DMF-T index greater than 4	1.09	1.00–1.19	0.044*

DMF-T index: Number of decayed, missing or filled teeth; PR: prevalence ratio; 95%CI: 95% confidence interval.

The dataset of this study is available in “Repositório USP” at https://repositorio.uspdigital.usp.br/handle/item/384.

## Discussion

We observed a high prevalence of gingivitis and calculus in a representative sample of 12-year-old schoolchildren from Quito, Ecuador. BOP and dental calculus prevalence were 92% and 69.9%, respectively.

The poor periodontal condition observed in this survey is comparable with a previous epidemiological study performed with 1070 high school adolescents from Santiago de Chile (Chile), Buenos Aires, Córdoba, Mendoza (Argentina), Montevideo (Uruguay), Quito (Ecuador), and Medellin (Colombia) that revealed that the worst periodontal conditions were verified in Quito compared to all other cities. Living in Quito was significantly associated with having at least one site with CAL ≥ 3 mm (OR = 520.2). Moreover, CAL ≥ 3 mm in ≥ 1 sites was significantly more prevalent in those with a BOP ≥25%.^9^

Almost all schoolchildren examined in our study had at least one sextant with BOP. This observation is consistent with the results of other studies conducted in Latin America, which observed that BOP is highly prevalent in the young population.^14-21^ In contrast, other cross-sectional studies performed in Latin American countries revealed a lower prevalence of gingival bleeding.^22-26^ The prevalence of gingivitis in Latin American populations varies widely. A review calculated the adjusted prevalence estimate of gingivitis in children and adolescents of Latin American countries, by including the total number of subjects and those with gingivitis from at least one representative study in each country. The adjusted prevalence was 34.7%, with the highest prevalence found in Colombia (77%) and Bolivia (73%) and the lowest prevalence in Mexico (23%). Calculus prevalence also varies widely in Latin America.^3^ Higher^21,27^and lower^25,28,29^prevalence rates were found in different studies and countries. These discrepancies could be explained by the heterogeneity of population characteristics and cultures. Further, differences in the examination protocol (index vs. partial recording vs. full mouth), number and calibration of examiners, type of area (urban vs. rural), and type of school (public vs. private) may have accounted for these differences, as access to oral health services and hygiene practices may differ between these settings.^3^

In the studies performed in Bolivia^30^ and Colombia,^31^ lack of oral health education was identified as a predisposing factor. This suggests that a lack of knowledge and awareness about proper oral hygiene practices contributes to the higher prevalence of gingival bleeding in the population. The results from our study align with previous research, emphasizing the importance of education, oral hygiene practices, and other socioeconomic factors in the oral health outcomes of children and adolescents.

Overall, the wide variability in the prevalence of gingivitis and calculus in Latin America underscores the importance of continued research and tailored interventions to improve oral health outcomes in this region. By understanding the local prevalence and factors contributing to poor oral health, targeted and effective interventions can be developed to address these issues.

In our study, lower levels of maternal education were significantly associated with BOP and calculus. Schoolchildren whose mothers had a higher education than primary education had a 5% lower prevalence of BOP compared to those whose mothers had completed primary school. Interestingly, father’s schooling was not significantly associated with the outcome in the multiple model, possibly due to collinearity with mother’s schooling. There is strong evidence indicating that shorter periods of formal maternal education are associated with worse oral health parameters in children.^20,30,31,32^ This association might occur because schoolchildren whose mothers have limited education tend to have fewer cognitive or socioemotional skills and lower school achievement than their counterparts.^31^Moreover, this variable is generally related to socioeconomic levels. The association between maternal education and BOP suggests that educational interventions may be effective in reducing the prevalence of periodontal disease in this population.

Furthermore, a significant association was observed between BOP and malocclusion. Schoolchildren with severe or handicapping malocclusion had a 10% higher prevalence of gingival bleeding compared to those with normal occlusion. This association was also verified in other studies.^33,34,35^ This finding may be explained by the fact that malpositioned teeth might favor plaque accumulation and consequently result in periodontal inflammation.^36^

The strengths of our study are the population-based survey data with a representative sample, an adequate sample size, and a high response rate, which minimizes the chance for random errors. Likewise, the rigorous examination protocol and repeated calibration training of examiners ensured a high reproducibility of registered data.

The study’s main limitation was the use of CPI to measure periodontal disease. CPI does not consider periodontal attachment loss, and using index teeth may underestimate the extent and severity of periodontal destruction.^37^ However, this is the most used index in epidemiological studies, and it is recommended by the WHO.^12^ Although the CPI index was used to examine periodontal parameters, BOP and presence of calculus were registered separately in our study. Another limitation of our study is that only students from public schools were included in this study. This might have overestimated the periodontal parameters, considering that private school students have milder periodontal conditions.^19,21,27^

High percentages of gingivitis and calculus were observed in this population. Preventive actions and early treatment could improve the periodontal parameters in children and adolescents of Quito, Ecuador. Oral health policies should focus on schoolchildren’s periodontal health and reinforce oral health services. Health promotion and education should be the main strategies for improving oral hygiene. Oral hygiene habits acquired at a young age usually continue throughout adulthood.^38^ As gingivitis is a reversible condition, appropriate treatment as soon as the disease is detected could prevent the development of periodontitis and its potential systemic complications in the future.^14^

Future prospective studies should investigate the determinants of the high prevalence of gingival bleeding and calculus in Quito. Further, full-mouth periodontal examination, pocket depth, and CAL could also be performed for a more accurate diagnosis of periodontal conditions in this population.

## Conclusion

Gingivitis and calculus are highly prevalent in schoolchildren from Quito. Gingival bleeding is associated with mother education and malocclusion, and dental calculus is associated with mother education and dental caries.
